# A Novel Prescribed-Performance-Tracking Control System with Finite-Time Convergence Stability for Uncertain Robotic Manipulators

**DOI:** 10.3390/s22072615

**Published:** 2022-03-29

**Authors:** Anh Tuan Vo, Thanh Nguyen Truong, Hee-Jun Kang

**Affiliations:** 1 Department of Electrical, Electronic and Computer Engineering, University of Ulsan, Ulsan 44610, Korea; voanhtuan2204@gmail.com (A.T.V.); thanhnguyen151095@gmail.com (T.N.T.); 2 School of Electrical Engineering, University of Ulsan, Ulsan 44610, Korea

**Keywords:** sliding mode control, finite-time control, robot manipulators, prescribed performance control

## Abstract

Through this article, we present an advanced prescribed performance-tracking control system with finite-time convergence stability for uncertain robotic manipulators. It is therefore necessary to define a suitable performance function and error transformation to guarantee a prescribed performance within a finite time. Following the definitions mentioned, a modified integral nonlinear sliding-mode hyperplane is constructed from the transformed errors. By using the designed nonlinear sliding-mode surface and the super-twisting reaching control law, an advanced approach to the prescribed performance control was formed for the trajectory tracking control of uncertain robotic manipulators. The proposed controller exhibits improved properties, including estimated convergence speed and a predefined upper and lower limit for maximum overshoot during transient responses. Furthermore, the maximum allowable size of the control errors at the steady-state can be predefined and these errors will inevitably converge to zero within a finite time, while the proposed controller can provide a smooth control torque without the loss of its robustness. It is shown that the proposed control system is globally stable and convergent over a finite time. A comprehensive analysis of the effectiveness of the proposed control algorithm was already conducted via the simulation of an industrial robot manipulator.

## 1. Introduction

In spite of the wide usage of robot manipulators in the real world, motion control of the manipulators with high precision remains an important issue due to the effects of unmodeled uncertainties, high nonlinearity, coupling dynamics, uncertain dynamics, and parametric variations [1]. To obtain high tracking accuracy and stability of control performance, the given control solutions should take into account the above-mentioned factors, as well as external disturbances or sensor noise from the environment, etc. However, those elements are never known with absolute certainty in reality. One of the most basic tasks in the robot control world is controlling the robot manipulators to exactly follow a prescribed trajectory. In terms of practical considerations, achieving high tracking accuracy and ensuring finite-time convergence for stable equilibrium in the presence of the noted elements is a desirable control target. With such a purpose, SMC [2,3] seems to be the best technique in the face of uncertainty. Nevertheless, the primary weakness of the conventional FOSMC is mainly associated with the chattering phenomenon [4]; moreover, it provides one-sliding accuracy [5]. To eliminate chattering effects, SOSMC and HOSMC were proposed, and their applications have been carefully analyzed in [6,7]. Unfortunately, the strategies mentioned in [6,7] are only appropriate to dynamic systems with a single input. Several recent control methods, such as fuzzy logic controllers [8,9], fault-tolerant controller [10], TSMCs [11,12] and FnTC [13,14,15,16] have been introduced. The outstanding features of such controllers include the ability to reject disturbances, robustness, finite-time, and fast convergence. Therefore, several FnTC strategies with good features have also been extended to industrial robots in recent times in order to address stabilization problems and/or tracking problems [6,15,17,18,19]. Although the mentioned methods are good, they still suffer from some shortcomings that need to be overcome in reality. The problem with Aghababa’s methods [13,14] is that, for example, the method [14] demands two discontinuous control signals that result in chattering behavior. Therefore, it cannot provide optimal control performance for controlled systems. The result of chattering, which appears as dangerous system vibrations, is that moving parts in actuators and mechanical actuators are often subjected to unnecessary wear and tear [20]. The control design strategy presented in [21] addresses the unpredictable chattering phenomenon directly and achieves a high level of performance. The above method was developed according to the STM, which efficiently alleviates the undesired chattering behavior and maintains the high precision for sliding-mode-based control algorithms [22,23].

Stabilization and tracking issues of the controlled systems are other important problems that we need to consider thoroughly. In spite of the fact that the above controllers can drive the state error variables to decrease to zero as the time approaches infinity and to converge to zero in a finite time, it is also not easy to control the transient responses that maintain in a limited region directly. A proposal in [24] referred to as prescribed performance sought to ensure that the controlled error variable can be limited in an arbitrarily small region so long as maximum overshoot is lower than a predefined constant and convergence speed obtains faster than a predefined value. With the concept of prescribed performance, the problems of FnTC have recently been solved with a combination of PPC and TSMC [25] or BsCM. Using a PPF, a hybrid tracking controller was developed in [26] based on FnTC and adaptive control for non-strict feedback systems. A BsCM was proposed in [27] based on the concept of a finite-time performance function. Basically, this means that after a finite time, these two methods are able to achieve finite-time stability, which means that the tracking errors will only be bounded after a finite time. Therefore, tracking errors under both control methods will not be able to converge to the exact origin within a finite time. Another BsCM for second-order systems has been introduced using a complex sliding surface to obtain fixed-time tracking [28]. This work yields the practical stability of fixed-time tracking, i.e., once the fixed-time stability is reached, tracking errors can only enter neighborhoods of zero.

In light of the previous discussions, this article proposes an advanced method to control the trajectory states of URMs. Our achievements in the present study are as follows:

- We avoid any singularity problems by providing a modified integral nonlinear sliding-mode hyperplane.
- The improved control scheme is built on a combination of the STM [21], the designed nonlinear sliding-mode surface, and a performance function concept [24]. As a result, the desirable transient state is maintained, the maximum allowable size of the control errors at the steady-state can be predefined, and these errors will inevitably converge to origin within a finite time.
- With a PPF, one can manage the convergence rate and the maximum overshoot of the state error variables during the transient response process; furthermore, a modified integral nonlinear sliding-mode hyperplane combined with the STM allows for substantially reducing all chattering effects and finally achieving high tracking accuracy for state error variables in a steady-state response.
- The rigorous investigation provides sufficient evidence of stability and convergence in a finite time.
- A full evaluation of the effectiveness of the proposed control algorithm and a comparison of its performance to that of other control methods have already been conducted via simulation of an industrial robot manipulator.

The following is the arrangement of the article. In the next section, we introduce the relevant preliminaries and formulate the considered statement. A description of the formulation of the developed control system and the analysis of the whole system’s stability is presented in Section 3. Section 4 examines the control performance of the proposed control strategy using a 3-DoF industrial robot system subjected to uncertain terms. Finally, Section 5 summarizes some of the important conclusions gained from the simulation results and the proposed theory.

## 2. Basic Definitions, Lemmas, and Problem Statement

### 2.1. Basic Definitions and Lemmas

To solve the problem and prove the convergence and stability of the proposed control algorithm, mathematical statements, assumptions, lemmas, and definitions are required.

The following expression describes the sgn(·) function:

(1) $$\begin{array}{c} {\left\lceil x\right\rceil }^{0}=\mathrm{sgn}\left(x\right)=\left\{\begin{array}{c} 1\;\;\;\mathrm{if}\;\;x>0\\ 0\;\;\mathrm{if}\;\;x=0\\ -1\;\;\mathrm{otherwise} \end{array}\right.. \end{array}$$

An obvious fact that can be proved for φ≥0,

(2) $$\begin{array}{c} {\left\lceil x\right\rceil }^{\varphi }={\left|x\right|}^{\varphi }\mathrm{sgn}\left(x\right) \end{array}$$

Taking the nonlinear system of the form as an example:

(3) $$\begin{array}{c} \dot{x}=f\left(x,t\right),\;x_{0}=x\left(0\right), \end{array}$$

where *f*: $R^{n}\times R_{+}\to R^{n}$, and let us assume that f(0,t)=0. The solutions of Equation (3) are understood in the sense of Filippov.

**Definition** **1** ([29]). *A solution* $x(x_{0},t)$ *of Equation (3) reaches equilibrium in some finite time moment if the origin of Equation (3) is globally asymptotically stable, i.e.,* $x(x_{0},t)=0$*,* $\forall t\geq T(x_{0})$*, where T:* $R^{n}\to R_{+}\cup \left\{0\right\}$ *is the settling-time function; a system such as Equation (3) is referred to as global finite-time stability around its equilibrium at the origin.*

**Definition** **2** ([24]). *In the case of a smooth function ψ:* $R_{+}\to R_{+}$*, the following properties are presented:*

-
$\lim_{t\to \infty }\psi \left(t\right)=\psi_{\infty }>0$ *;*
-
ψ(t) *is decreasing and positive;*

*In this case, it is considered to be a performance function.*

**Lemma** **1** ([30]). *The differential equation has the following origin:*

(4) $$\begin{array}{c} {\left\lceil q^{\left(j\right)}\right\rceil }^{\frac{\theta }{h-j}}+\rho_{j-1}\left\{{\left\lceil q^{\left(j-1\right)}\right\rceil }^{\frac{\theta }{h-j+1}}\left.+\ldots +\rho_{2}\left[{\left\lceil \ddot{q}\right\rceil }^{\frac{\theta }{h-2}}+\rho_{1}\left({\left\lceil \dot{q}\right\rceil }^{\frac{\theta }{h-1}}+\rho_{0}{\left\lceil q\right\rceil }^{\frac{\theta }{h}}\right)\right]\ldots \right\}=0\right., \end{array}$$

*This equation is finite-time stable for each j=1,…,h−1; in that case, it satisfies the following condition where $\rho_{k}$, (k=0,…,h−1) are chosen to be sufficient, h≥2 is an integer, and θ is a positive scalar.*

**Lemma** **2** ([21]). *Let us consider the system as follows:*

(5) $$\begin{array}{c} \left\{\begin{array}{c} \dot{\varpi }=-\alpha_{1}\left(t\right){\left\lceil \varpi \right\rceil }^{1/2}-\alpha_{2}\left(t\right)\varpi +\gamma \\ \dot{\gamma }=-\alpha_{3}\left(t\right){\left\lceil \varpi \right\rceil }^{0}-\alpha_{4}\left(t\right)\varpi +\chi \left(t\right) \end{array}\right.. \end{array}$$

*It is assumed that there exists some unknown scalar $\delta_{\chi }\geq 0$ such that $\left|\chi (t)\right|\leq \delta_{\chi }$. If $\phi_{0}\left(t\right)$ is a positive function, it has derivative with respect to time, as follows:*

(6) $$\begin{array}{c} {\dot{\phi }}_{0}\left(t\right)=\left\{\begin{array}{c} \varepsilon \;\;\;\mathrm{if}\;\;\;\left|\varpi \right|\geq \delta_{\varpi }\\ 0\;\;\;\mathrm{otherwise} \end{array}\right., \end{array}$$

*where $\delta_{\varpi }$ is defined as an arbitrary positive scalar, and the gains with respect to time-varying $\alpha_{m}\left(t\right)$, (m=1,2,3,4) can be obtained from:*

(7) $$\begin{array}{c} \begin{array}{c} \alpha_{1}\left(t\right)=\alpha_{10}\sqrt{\phi_{0}\left(t\right)};\alpha_{3}\left(t\right)=\alpha_{30}\phi_{0}\left(t\right);\\ \alpha_{2}\left(t\right)=\alpha_{20}\phi_{0}\left(t\right);\pi_{4}\left(t\right)=\alpha_{40}\phi_{0}^{2}\left(t\right), \end{array} \end{array}$$

*in which positive constants $\alpha_{m0}$ satisfy*

(8) $$\begin{array}{c} 4\alpha_{30}\alpha_{40}\geq \left(8\alpha_{30}+9\alpha_{10}^{2}\right)\alpha_{20}^{2}. \end{array}$$

*Therefore, the state variables of Equation (5) converge to the exact origin within a finite time.*

### 2.2. Dynamic Modeling of Robotic Manipulators

Dynamic modeling of URMs is described by the following form [31]:

(9) $$\begin{array}{c} M\left(p\right)\ddot{p}+C\left(p,\dot{p}\right)\dot{p}+G\left(p\right)+f_{r}\left(\dot{p}\right)=\tau -\tau_{d}\left(t\right), \end{array}$$

where $p\in R^{n\times 1}$, $\dot{p}\in R^{n\times 1}$, and $\ddot{p}\in R^{n\times 1}$ represent vector of joint angular position, vector of joint angular velocity, and vector of joint angular acceleration, respectively. $M\left(p\right)=\hat{M}\left(p\right)+\Delta M\left(p\right)\in R^{n\times n}$, $C\left(p,\dot{p}\right)=\hat{C}\left(p,\dot{p}\right)+\Delta C\left(p,\dot{p}\right)\in R^{n\times n}$, and $G\left(p\right)=\hat{G}\left(p\right)+\Delta G\left(p\right)\in R^{n\times 1}$ represent positive–definite and symmetric matrix of inertia parameters, matrix of the Coriolis and centripetal forces, and vectors of the gravitational force, respectively. $\hat{M}\left(p\right)\in R^{n\times n}$, $\hat{C}\left(p,\dot{p}\right)\in R^{n\times n}$, and $\hat{G}\left(p\right)\in R^{n\times 1}$ represent the estimated part of $M\left(p\right)$, the estimated part of $C\left(p,\dot{p}\right)$, and the estimated part of $G\left(p\right)$, respectively. $\Delta M\left(p\right)\in R^{n\times n}$, $\Delta C\left(p,\dot{p}\right)\in R^{n\times n}$, and $\Delta G\left(p\right)\in R^{n\times 1}$ represent the uncertain dynamic part of $M\left(p\right)$, the uncertain dynamic part of $C\left(p,\dot{p}\right)$, and the uncertain dynamic part of $G\left(p\right)$, respectively. $f_{r}\left(\dot{p}\right)\in R^{n\times 1}$, $\tau_{d}\left(t\right)\in R^{n\times 1}$, and $\tau \in R^{n\times 1}$ represent vectors of the friction force, unknown time-varying external disturbance, and the vector of the control input torque, respectively.

Let us define the state vector and the control input torque vector, respectively, by $x={\left[x_{1},x_{2}\right]}^{T}={\left[p,\dot{p}\right]}^{T}$ and u=τ. Consequently, the dynamic model (9) is described in the form of second-order nonlinear systems, as follows:

(10) $$\begin{array}{c} \left\{\begin{array}{c} {\dot{x}}_{1}=x_{2}\\ {\dot{x}}_{2}=Z\left(x\right)u+A\left(x\right)+\delta \left(x,\Delta ,\tau_{d}\right) \end{array}\right., \end{array}$$

where $A\left(x\right)=-{\hat{M}}^{-1}\left(p\right)\left(\hat{C}\left(p,\dot{p}\right)\dot{p}+\hat{G}\left(p\right)\right)$, $Z\left(x\right)={\hat{M}}^{-1}\left(p\right)$ and $\delta \left(x,\Delta ,\tau_{d}\right)=-{\hat{M}}^{-1}\left(p\right)\times \left(f_{r}\left(\dot{p}\right)+\Delta M\left(p\right)\ddot{p}+\Delta C\left(p,\dot{p}\right)\dot{p}+\Delta G\left(p\right)+\tau_{d}\left(t\right)\right)$ represent smooth nonlinear functions and the lumped undefined uncertainty, respectively.

As the title of our paper implies, we propose an improved method of controlling the trajectory states of URMs to ensure that they follow a specified trajectory precisely within a finite time. Moreover, convergence rate, transient, and steady-state response are within a pre-specified performance boundary.

**Assumption** **1.** *For controls, the trajectory states of Equation (10), the desired trajectory $x_{d}\left(t\right)$, and their higher-order derivatives are bounded for all time.*

## 3. Design of the Proposed Control Method

### 3.1. Prescribed Performance Function

In this subsection, a PPF will be used to reconstruct the trajectory tracking errors of the URMs.

The vector of trajectory tracking error is defined as follows:

(11) $$\begin{array}{c} e\left(t\right)={\left[e_{1},e_{2}\right]}^{T}={\left[x_{1}-x_{d}\left(t\right),x_{2}-{\dot{x}}_{d}\left(t\right)\right]}^{T}. \end{array}$$

Then, a positive decreasing smooth function ψ: $R_{+}\to R_{+}$ is introduced as a PPF [24]:

(12) $$\begin{array}{c} \psi \left(t\right)=\left(\psi_{0}-\psi_{\infty }\right)\epsilon^{-\mu t}+\psi_{\infty }, \end{array}$$

where ϵ is Euler’s number. $\psi_{0}>\psi_{\infty }>0$ and μ>0 are user-designed parameters.

The following condition allows the trajectory tracking error to be maintained within the specified range below:

(13) $$\begin{array}{c} -\delta_{l}\psi \left(t\right)<e\left(t\right)<\delta_{u}\psi \left(t\right),\;\;\forall t>0, \end{array}$$

where $\delta_{l}$ and $\delta_{u}$ are positive parameters.

**Remark** **1.** *In Equation (13), $-\delta_{l}\psi \left(t\right)$ and $\delta_{u}\psi \left(t\right)$ represent the lower bound of the undershoot and the upper bound of the maximum overshoot. μ denotes the convergence speed and $\psi_{\infty }$ defines the steady-state tracking error. As such, tuning the parameters such as $\delta_{l},\delta_{u},\mu ,\psi_{0}$, and $\psi_{\infty }$, to optimize steady-state and transient performance is possible.*

For designing the PPC, the transformed error $s_{1}\in R$ is defined based on [24] as a smooth, strictly increasing function $\sigma (s_{1})$ such that:

$-\delta_{l}<\sigma \left(s_{1}\right)<\delta_{u},\;\;\forall s_{1}\in L_{\infty }$

$\lim_{s_{1}\to +\infty }\sigma \left(s_{1}\right)=\delta_{u}$, and $\lim_{s_{1}\to -\infty }\sigma \left(s_{1}\right)=-\delta_{l}$.

The error transformation is applied from the properties of $\sigma (s_{1})$ as:

(14) $$\begin{array}{c} e\left(t\right)=\psi \left(t\right)\sigma \left(s_{1}\right). \end{array}$$

Since $\sigma (s_{1})$ is strictly monotonic increasing and $\psi \left(t\right)\geq \psi_{\infty }>0$, the transformed error $s_{1}$ can be obtained from the inverse function of $\sigma (s_{1})$ as:

(15) $$\begin{array}{c} s_{1}=\sigma^{-1}\left[\frac{e(t)}{\psi (t)}\right]. \end{array}$$

The design parameters $\delta_{l},\delta_{u},\mu ,\psi \left(0\right)$, $\psi_{\infty }$ of Equations (12) and (13), and function $\sigma (s_{1})$, can be chosen. For any initial condition e(0), if $\delta_{l}$, $\delta_{u}$, and ψ(0) are selected to satisfy the condition $-\delta_{l}\psi \left(0\right)<e\left(0\right)<\delta_{u}\psi \left(0\right)$, then $z_{1}$ is bounded, and the condition $-\delta_{l}<\sigma \left(s_{1}\right)<\delta_{u}$ holds. Therefore, the condition in Equation (13) is always satisfied, with both transient and steady-state responses being guaranteed for pre-determined behavior boundaries.

Selecting the function $\sigma (s_{1})$ as:

(16) $$\begin{array}{c} \sigma \left(s_{1}\right)=\frac{\delta_{u}\epsilon^{s_{1}}-\delta_{l}\epsilon^{-s_{1}}}{\epsilon^{s_{1}}+\epsilon^{-s_{1}}}. \end{array}$$

Since the transformed error $s_{1}$ is bounded, it is computed by:

(17) $$\begin{array}{c} s_{1}=\sigma^{-1}\left[\frac{e(t)}{\psi (t)}\right]=\frac{1}{2}\ln\frac{\varepsilon \left(t\right)+\delta_{l}}{\delta_{u}-\varepsilon \left(t\right)}, \end{array}$$

where $\varepsilon \left(t\right)=\frac{e(t)}{\psi (t)}$ is defined as the normalized tracking error.

### 3.2. Change of Coordinates

Taking the first and second derivative of $s_{1}$ and using the normalized tracking error yields, respectively:

(18) $$\begin{array}{c} {\dot{s}}_{1}=\xi \left(\dot{e}-\frac{e\dot{\psi }}{\psi }\right), \end{array}$$

(19) $$\begin{array}{c} {\ddot{s}}_{1}=\dot{\xi }\vartheta_{1}\left(e,t\right)+\xi \left(\ddot{e}+\vartheta_{2}\left(e,t\right)\right), \end{array}$$

where $\xi \left(e,t\right)=\frac{1}{2\psi }\left(\frac{1}{\varepsilon +\delta_{l}}-\frac{1}{\varepsilon -\delta_{u}}\right)$, $0<\xi <\xi_{M}$, $\vartheta_{1}\left(e,t\right)=\left(\dot{e}-\frac{e\dot{\psi }}{\psi }\right)$, $\vartheta_{2}\left(e,t\right)=-\frac{\dot{e}\dot{\psi }}{\psi }-\frac{e\ddot{\psi }}{\psi }+\frac{e{\dot{\psi }}^{2}}{\psi^{2}}$, and $\dot{\xi }=-\frac{\dot{\psi }}{2\psi^{2}}\left(\frac{1}{\varepsilon +\delta_{l}}-\frac{1}{\varepsilon -\delta_{u}}\right)-\frac{\dot{e}\psi -e\dot{\psi }}{2\psi^{3}}\left(\frac{1}{{\left(\varepsilon +\delta_{l}\right)}^{2}}-\frac{1}{{\left(\varepsilon -\delta_{u}\right)}^{2}}\right)$.

Noting Equations (10), (18) and (19), dynamic modeling of URMs can now be described in the following compact form:

(20) $$\begin{array}{c} \left\{\begin{array}{c} {\dot{s}}_{1}=s_{2}\\ {\dot{s}}_{2}=\dot{\xi }\vartheta_{1}\left(e,t\right)+\xi \left(Z\left(x\right)u+\vartheta_{2}\left(e,t\right)-{\ddot{x}}_{d}+A\left(x\right)+\delta \left(x,\Delta ,\tau_{d}\right)\right) \end{array}\right.. \end{array}$$

**Assumption** **2** ([32]). *$\xi \delta_{i}\left(x,\Delta ,\tau_{d}\right)$ represents the undefined uncertain terms which are a Lipschitz continuous function with respect to time-varying; hence it follows that:*

(21) $$\begin{array}{c} \left|\xi {\dot{\delta }}_{i}\left(x,\Delta ,\tau_{d}\right)\right|<{\bar{\delta }}^{*}, \end{array}$$

where ${\bar{\delta }}^{*}$ is a positive constant.

### 3.3. Proposing of a Modified Integral Nonlinear Sliding-Mode Hyperplane

Based on the transformed error, we propose a modified integral nonlinear sliding-mode hyperplane designed to remove singularity problems and guarantee a sliding-mode motion in a finite time:

(22) $$\begin{array}{c} S=s_{2}-s_{2}\left(0\right)+\int_{0}^{t}{\left\lceil \rho_{1}\left({\left\lceil s_{2}\right\rceil }^{\frac{3}{2}}+\rho_{0}s_{1}\right)\right\rceil }^{\frac{1}{3}}d\iota , \end{array}$$

where ι is the time variable, *S* is a sliding-mode hyperplane, and $\rho_{0}$ and $\rho_{1}$ are user-designed coefficients.

As long as the proposed system works in sliding mode, S=0 and $\dot{S}=0$, then it is considered to be in sliding mode. As a result, Equation (22) yields:

(23) $$\begin{array}{c} {\dot{s}}_{2}=-{\left\lceil \rho_{1}\left({\left\lceil s_{2}\right\rceil }^{\frac{3}{2}}+\rho_{0}s_{1}\right)\right\rceil }^{\frac{1}{3}}, \end{array}$$

and taking Equation (20) into account, it follows that:

(24) $$\begin{array}{c} \left\{\begin{array}{c} {\dot{s}}_{1}=s_{2}\\ {\left\lceil {\ddot{s}}_{1}\right\rceil }^{3}+\rho_{1}\left({\left\lceil s_{2}\right\rceil }^{\frac{3}{2}}+\rho_{0}s_{1}\right)=0 \end{array}\right.. \end{array}$$

The differential Equation (4) essentially becomes the differential Equation (24) with θ=h=3 and j=2. For any initial state vectors, $\left[s_{0}\right]$, the system states, s(t), converge to the exact origin within a finite time according to Lemma 1.

### 3.4. Proposed Controller Design

Based on the proposed integral nonlinear sliding-mode hyperplane, as proposed in Equation (22), this subsection is dedicated to designing the suitable control laws to take place the finite-time sliding motion. The synthesis of the proposed controller is accomplished in the following sequence.

Calculating the time-related derivative for the sliding-mode hyperplane (22) while noting the dynamic system (20), we obtain:

(25) $$\begin{array}{c} \dot{S}=\dot{\xi }\vartheta_{1}\left(e,t\right)+{\left\lceil \rho_{1}\left({\left\lceil s_{2}\right\rceil }^{\frac{3}{2}}+\rho_{0}s_{1}\right)\right\rceil }^{\frac{1}{3}}+\xi \left(Z\left(x\right)u+\vartheta_{2}\left(e,t\right)-{\ddot{x}}_{d}+A\left(x\right)+\delta \left(x,\Delta ,\tau_{d}\right)\right). \end{array}$$

Based on Dynamic (25), Lemma 2, and PPC, the proposed control law is designed as follows:

(26) $$\begin{array}{c} u=-Z^{-1}\left(x\right)\xi^{-1}\left(u_{eq}+u_{r}\right), \end{array}$$

where the equivalent term $u_{eq}$ is constructed without considering the lumped uncertain terms, as follows:

$$\begin{array}{c} \begin{array}{c} u_{eq}=\dot{\xi }\vartheta_{1}\left(e,t\right)+{\left\lceil \rho_{1}\left({\left\lceil s_{2}\right\rceil }^{\frac{3}{2}}+\rho_{0}s_{1}\right)\right\rceil }^{\frac{1}{3}}+\xi \left(A\left(x\right)-{\ddot{x}}_{d}+\vartheta_{2}\left(e,t\right)\right) \end{array}, \end{array}$$

and the reaching term is designed based on STM in Lemma 2, as follows:

$$\begin{array}{c} u_{r}=\alpha_{1}\left(t\right){\left\lceil S\right\rceil }^{\frac{1}{2}}+\alpha_{2}\left(t\right)S+\int_{0}^{t}\left[\alpha_{3}\left(t\right){\left\lceil S\right\rceil }^{0}+\alpha_{4}\left(t\right)S\right]d\iota . \end{array}$$

The control design process can be summarized in the following theorem.

**Theorem** **1.** *With the robot-system-satisfying Assumption 2, which is converted into the form of Equation (20) by using PPC, the proposed control system in Equation (26) based on an integral nonlinear sliding-mode hyperplane in Equation (22) and Lemma 2 provides the sliding-mode motion, i.e., S=0, that takes place in a finite time.*

**Proof of Theorem** **1.** With the proposed control law in Equation (26), Equation (25) becomes

(27) $$\begin{array}{c} \begin{array}{c} \dot{S}=\xi \delta \left(x,\Delta ,\tau_{d}\right)-u_{r}\\ \;\;\;\;\;=\xi \delta \left(x,\Delta ,\tau_{d}\right)-\alpha_{1}\left(t\right){\left\lceil S\right\rceil }^{\frac{1}{2}}-\alpha_{2}\left(t\right)S-\int_{0}^{t}\left[\alpha_{3}\left(t\right){\left\lceil S\right\rceil }^{0}+\alpha_{4}\left(t\right)S\right]d\iota \end{array}. \end{array}$$

We can see that the following whole terms can be considered a new term:

(28) $$\begin{array}{c} \gamma =-\int_{0}^{t}\left[\alpha_{3}\left(t\right){\left\lceil S\right\rceil }^{0}+\alpha_{4}\left(t\right)S\right]d\iota +\xi \delta \left(x,\Delta ,\tau_{d}\right). \end{array}$$

Therefore, Dynamic (27) is rewritten in the following expression:

(29) $$\begin{array}{c} \left\{\begin{array}{c} \dot{S}=-\alpha_{1}\left(t\right){\left\lceil S\right\rceil }^{\frac{1}{2}}-\alpha_{2}\left(t\right)S-\gamma \\ \dot{\gamma }=-\alpha_{3}\left(t\right){\left\lceil S\right\rceil }^{0}-\alpha_{4}\left(t\right)S+\xi \dot{\delta }\left(x,\Delta ,\tau_{d}\right) \end{array}\right.. \end{array}$$

According to Lemma 2 and Assumption 2, we infer that the state variables of Equation (29) will eventually converge to the exact origin, that is, S0= and γ=0 will be obtained within a finite time. □

**Figure 1:**
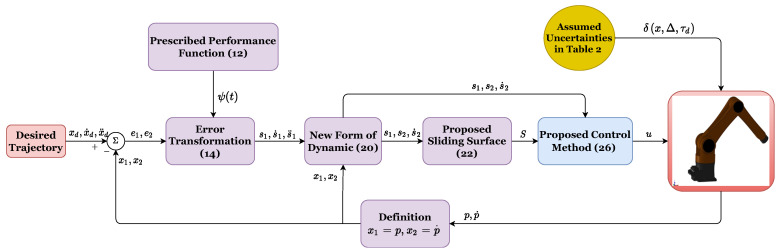
Algorithm diagram for the proposed control procedure.

The block diagram of the proposed control system can be found in Figure 1.

**Remark** **2.** *It is noted that the STM is a famous SOSM method which is essential to construct Lemma 2. With the S variable, the SOSM can therefore be obtained by proposing the modified sliding surface (22), where $S=\dot{S}=0$, which then leads to the $\left[s_{1}\right]\left(t\right)$ variable with a third-order sliding mode, i.e., $s_{1}=s_{2}={\dot{s}}_{2}=0,\left(r=3\right)$. Based on [5], the maximum possible sliding accuracy obtainable with a discrete switch is given by the relation of $\left|s_{1}\right|∼t_{s}^{r}$, with $t_{s}$ being the minimum switching interval. Therefore, the proposed controller can achieve its best possible accuracy with three-sliding accuracy.*

## 4. Simulation Results and Discussion

Simulations were performed using MATLAB/SIMULINK to evaluate aspects such as the management of maximum overshoot, convergence rate, and the transient and steady-state response. Furthermore, the evaluation of the chattering effects and tracking accuracy from the proposed controller are carefully considered via comparison to other similar methods such as SMC [2], TSMC [33], and FTSMC [33] for a 3-DoF robotic manipulator designed according to [31,34].

SMC based on [2] is typically constructed with a linear sliding surface such as:

(30) $$S_{0}=\dot{e}+c_{01}e,$$

in which $c_{01}$ stands for positive constants.

On the basis of [33], TSMC is developed with a nonlinear sliding surface such as:

(31) $$\begin{array}{c} S_{1}=\dot{e}+c_{11}{\left\lceil e\right\rceil }^{\kappa_{11}}, \end{array}$$

in which $c_{11}$ and $0<\kappa_{11}<1$ are positive constants.

The FTSMC is also based on [33] with a nonlinear sliding surface as follows:

(32) $$\begin{array}{c} S_{2}=\dot{e}+c_{21}e+c_{2}{\left\lceil e\right\rceil }^{\kappa_{22}}, \end{array}$$

in which $c_{21},c_{22}$, and $0<\kappa_{22}<1$ are positive constants.

**Remark** **3.** *The improved points related to the novelty of the proposed sliding surface can be stated as follows. It is constructed based on the transformed errors from error transformation and prescribed function of the prescribed performance control. Therefore, the desirable transient state is maintained, the maximum allowable size of the control errors at the steady state can be predefined, and these errors will inevitably converge to origin within a finite time. In methods including TSMC [33] and FTSMC [33], the sliding-mode hyperplane is constructed from the tracking error. They can obtain finite-time convergence or only asymptotic convergence. However, they cannot limit the convergence rate and the maximum overshoot of the state error. From Equations (31) and (32), the equivalent control input is constructed from ${\dot{S}}_{1}=0$ or ${\dot{S}}_{2}=0$. It can be seen that the first derivative of $S_{1}$ or $S_{2}$ includes $e^{\kappa_{11}-1}\dot{e}$ or $e^{\kappa_{22}-1}\dot{e}$, which has a finite escape time for the case of $\dot{e}\neq 0$ while e=0. Obviously, the proposed sliding surface does not contain such singularity problems. Further, the proposed sliding surface can easily be combined with STM to develop advanced control algorithms due to its structure.*

### 4.1. Configuration of the Testing System and Selection of Control Parameters

**Figure 2:**
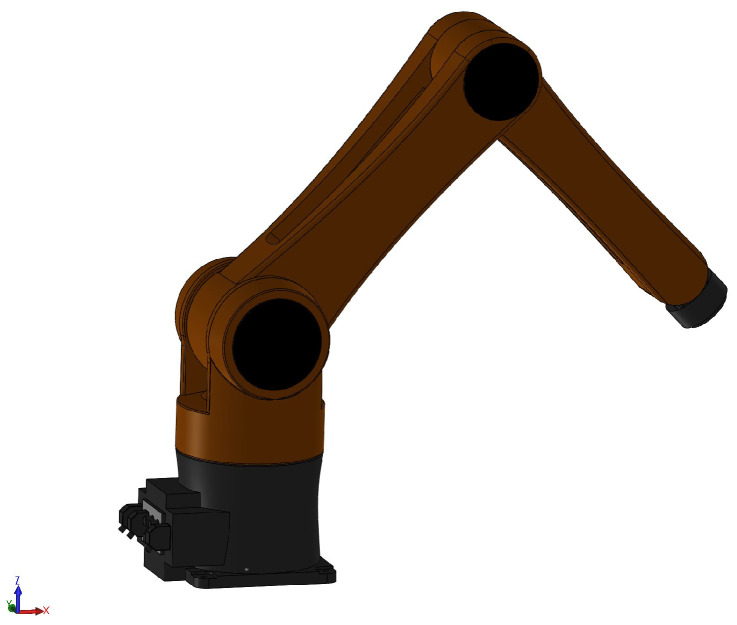
A 3-DoF robotic manipulator is described in a 3D CAD model by utilizing SOLIDWORKS and Simscape Multibody Link. Data from the study [35] were cited for the 3D CAD model of the robot.

A 3-DoF robotic manipulator was used as the testing system via a combination of MATLAB/SIMULINK and SOLIDWORKS for all simulation investigations. The dynamic calculations and kinematics of the robot are derived from [31,34]. The robot system has the parameters reported in Table 1. This robot was designed entirely in the SOLIDWORKS software, and includes all the mechanical parts such as links, joints, actuators, etc. In the SOLIDWORKS assembly environment, the robot parts were integrated with the coordinate system, the structure was completed, and the direction of the gravitational force was defined. Figure 2 shows a complete 3D representation of a 3-DoF robotic manipulator designed using SOLIDWORKS. In addition, SOLIDWORKS offers XML files and STEP files that can be exported using the Simscape Multibody Link tool. The robot’s mechanical components are completely described in the XML file, including mass, inertia moment, and the center of mass, as well as all parameters of the coordinate system as it relates to the assembly environment. In STEP files, the mechanical components are presented as 3D CAD models. In the end, the Simscape Multibody Link allows complete files to be embedded into MATLAB/SIMULINK to construct the control program for this robot system. This approach results in a robot model that is identical to the actual dynamic model. Measurement devices for position and velocity are based on the MATLAB/SIMULINK library. In the case of testing control performance, it is assumed that lumped uncertain terms including unknown dynamic models, disturbances, and friction will impact the system.

We solved the differential equations using Euler’s method with a sampling time of $t_{s}=10^{-3}$.

The robot manipulator’s task is to follow the specific trajectory shown below:

(33) $$\begin{array}{c} \left\{\begin{array}{c} x=0.85-0.01t\\ y=0.2+0.2\sin(0.5t)\\ z=0.7+0.2\cos(0.5t) \end{array}\right.\;\;\left(m\right). \end{array}$$

In all cases of the simulation, unknown uncertain terms such as dynamic uncertainties, frictions, and external disturbances were assumed in Table 2.

The selection of control parameters for the controller proposed in this paper is specifically guided in Remark 4.

**Remark** **4.** *To determine the lower bound of the undershoot, the upper bound of the maximum overshoot, and the convergence speed, and to optimize steady-state and transient performance, the parameters of the PPF are selected in the sense of Definition 2. From Equations (12) and (13), it is seen that $\psi_{0}$ is selected to be greater than the initial states and $\psi_{\infty }$ is the desired boundary of the steady-state tracking error. μ is tuned during the simulation calculation to obtain the desired convergence rate. Therefore, we selected $\delta_{l},\delta_{u},\mu ,\psi_{0}$, and $\psi_{\infty }$ as in Table 3. To be able to apply the results presented in Lemma 1, we then selected θ=h=3 and j=2; therefore, the differential equation as stated in Equation (4) essentially becomes the differential Equation (24). The parameters $\rho_{1},\rho_{2}$ of the proposed sliding-mode surface are selected as positive constants. The parameters of the reaching term, such as $\alpha_{1},\alpha_{2},\alpha_{3}$, and $\alpha_{4}$ in Equation (26), are selected based on the condition stated in Equation (8); therefore, we selected $\alpha_{10}=2,\alpha_{20}=6,\alpha_{30}=10$, and $\alpha_{40}=100$. The control parameters selected for the proposed control algorithm are described in Table 3. The parameters of the compared controllers are selected to achieve the best performance within their capabilities.*

### 4.2. Simulation Results and Discussion

**Figure 3:**
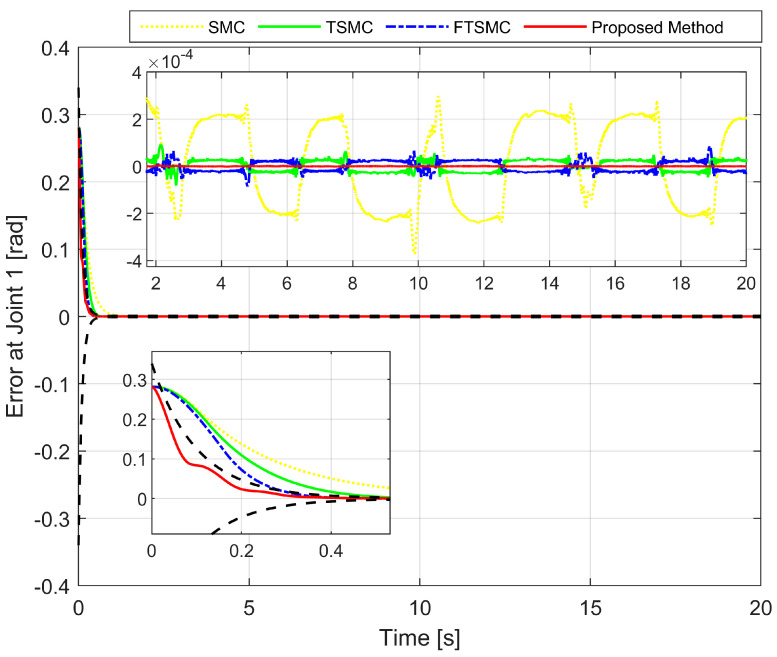
The first joint error comparison between the real trajectory of the robot and the reference trajectory.

**Figure 4:**
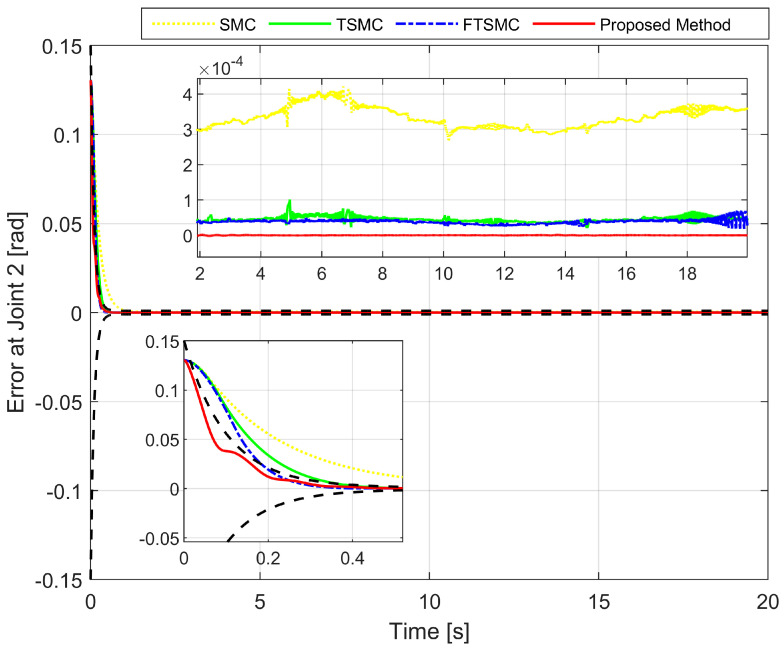
The second joint error comparison between the real trajectory of the robot and the reference trajectory.

**Figure 5:**
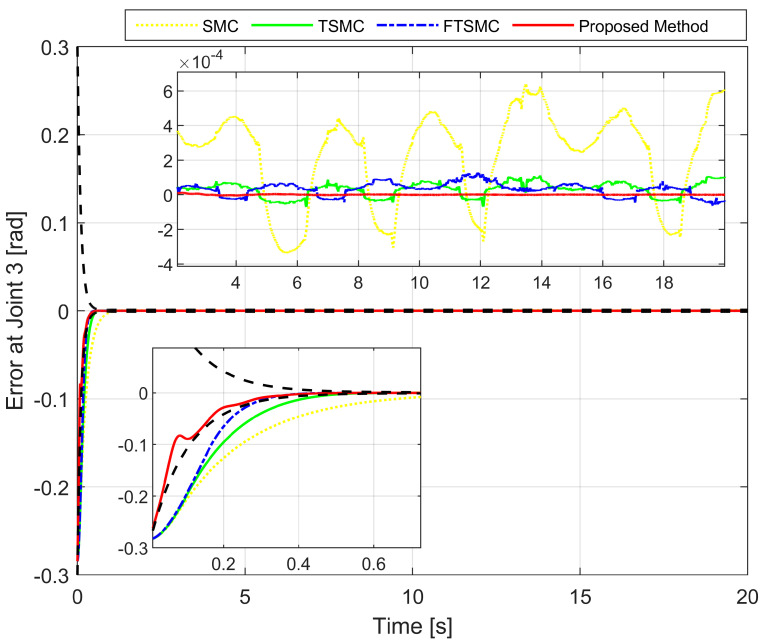
The third joint error comparison between the real trajectory of the robot and the reference trajectory.

We analyze the simulation results according to the regulation problem and the tracking problem. The regulation problem is analyzed through the results displayed in Figure 3, Figure 4 and Figure 5. All state error variables have the same initial value. However, the proposed controller exclusively provides a transient response within a specified performance. The state error variables are observed to satisfy the condition, as stated in Equation (13) which implies that we are able to manipulate both the convergence rate and the maximum overshoot of $e_{1}\left(t\right)$. We can tune the parameters such as $\delta_{l},\delta_{u},\mu ,\psi_{0}$, and $\psi_{\infty }$ to optimize the achievable steady-state and transient performance. In addition, the state error variables are also observed to converge to zero within a specified period of time. By contrast, the three remaining methods appear to make manipulating the transient response and maximum overshoot of $e_{1}\left(t\right)$ extremely difficult, as is clearly shown in the zoomed-in part of Figure 3, Figure 4 and Figure 5.

The tracking problem is considered in tracking a reference trajectory, given in Equation (33).

Position tracking errors are considered after the period of convergence time to equilibrium in order to analyze the accuracy of the tracking and facilitate performance evaluation. The investigation time ranged from 6 s → 20 s. Errors were determined by RMS and the results of the corresponding Equation (34) are shown in Table 4.

(34) $$E_{x}=\sqrt{\frac{1}{W}\sum_{i=1}^{W}{\left|\left(x_{ri}-x_{i}\right)\right|}^{2}};E_{y}=\sqrt{\frac{1}{W}\sum_{i=1}^{W}{\left|\left(y_{ri}-y_{i}\right)\right|}^{2}};E_{z}=\sqrt{\frac{1}{W}\sum_{i=1}^{W}{\left|\left(z_{ri}-z_{i}\right)\right|}^{2}},$$

in which *W* represents the number of samples taken into account. ${\left[x_{ri},y_{ri},z_{ri}\right]}^{T}$ and ${\left[x_{i},y_{i},z_{i}\right]}^{T}$ correspond to the desired end-effector position at time index *i* and the actual end-effector position at time index *i*, respectively.

**Figure 6:**
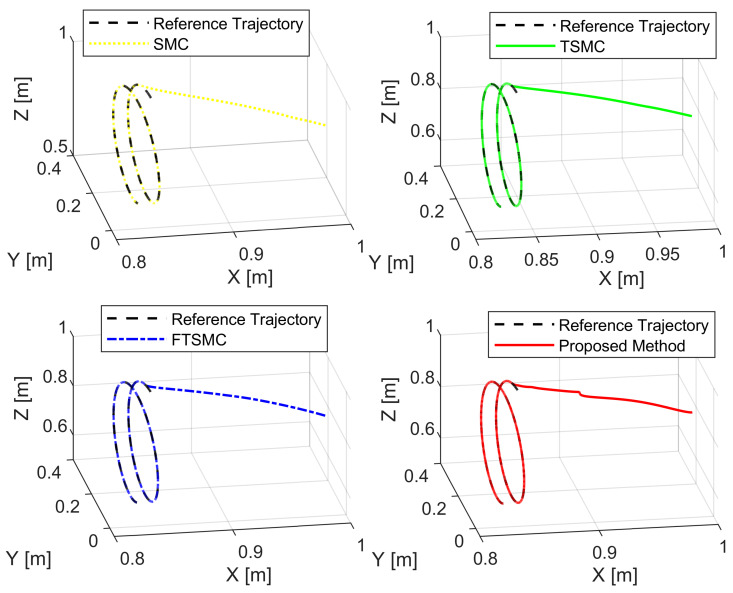
The real trajectory under each controller versus the desired trajectory.

**Figure 7:**
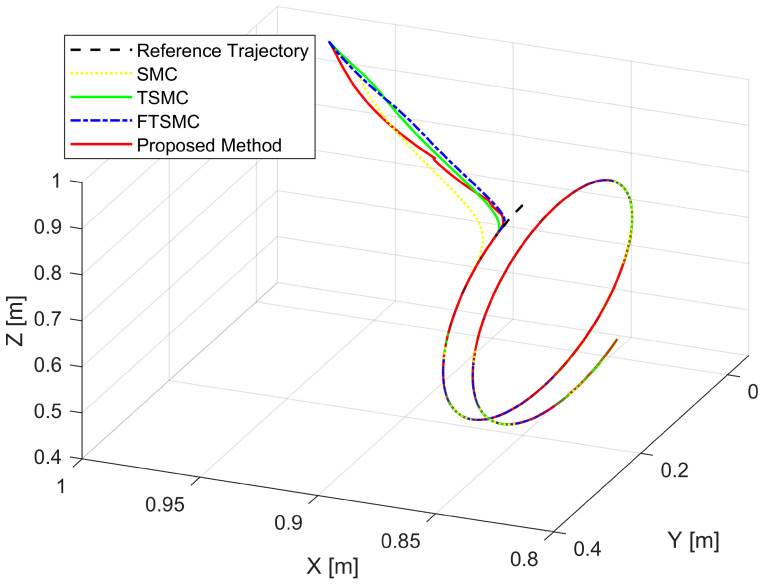
The real trajectories under all controllers versus the desired trajectory.

Figure 6 and Figure 7 show, respectively, the trajectory tracking curves of a 3-DoF manipulator under the control of four separate controllers. The real trajectories of the robot manipulator controlled by each controller seem to have matched the desired trajectory very well.

**Figure 8:**
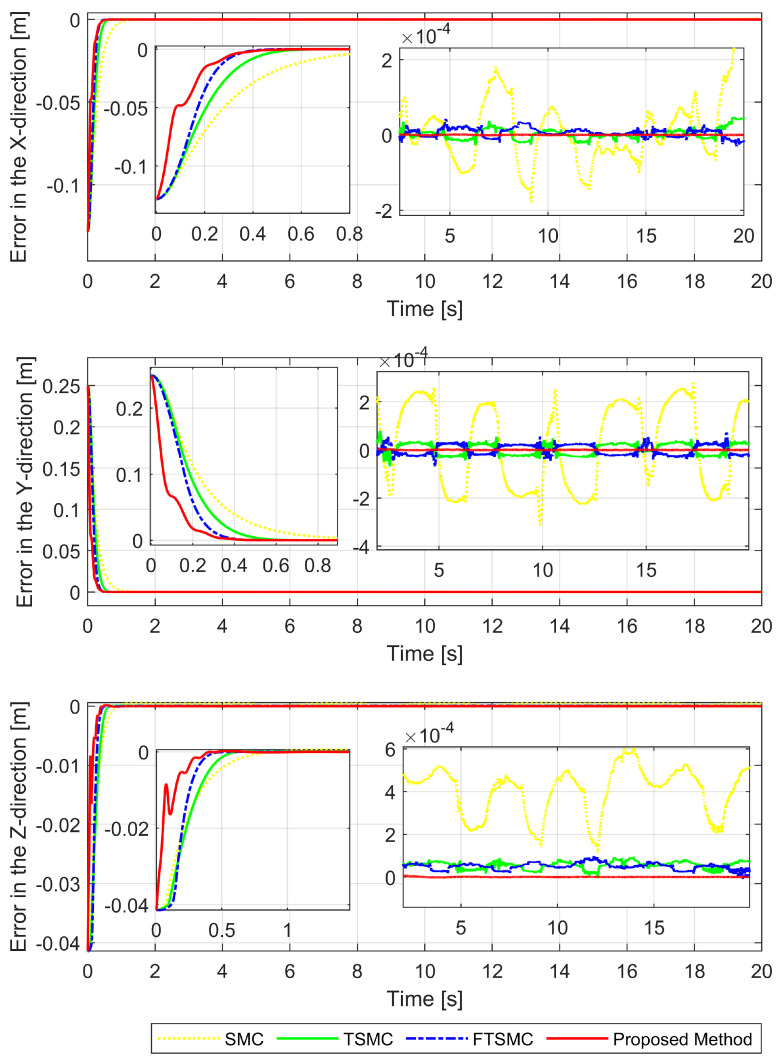
X-axis, Y-axis, and Z-axis error comparisons between the position of the end effector and the reference trajectory.

Figure 3, Figure 4 and Figure 5, respectively, show the tracking error comparison between the real trajectory of the robot and the reference trajectory at the three joints when suitable control parameters were chosen and the control performance from the controllers was relatively stable. Figure 8 shows X-axis, Y-axis, and Z-axis error comparisons between the position of the end effector and the reference trajectory.

From the mentioned figures, the first comparison in two features includes transient response and convergence speed among four controllers. We discovered that the proposed controller is the fastest. In general, faster transient response and faster convergence of state variables result in relatively larger control torques, thereby consuming more energy. While selecting control parameters for four separate controllers, trade-offs have to be performed among transient response, convergence speed, and the magnitude of the control input. Therefore, the control parameters were chosen so that the initial control torque would be as close as possible to the same while guaranteeing control efficiency. As shown in Figure 3, Figure 4 and Figure 5, the convergence speed and the steady-state error of the trajectory tracking error from the proposed controller can be precisely limited within the required preset performance, whereas the convergence speed and the tracking errors from the remaining three controllers are out of the preset boundaries (a predefined upper and lower limit for maximum overshoot) during transient responses. Our results indicate that the prescribed performance played an important role in controlling the transient response process, thus assigning convergence speed and limiting the maximum overshoot of the steady-state errors.

In a comparison of tracking accuracy using RMS level for X-axis, Y-axis, and Z-axis error, we saw that the proposed controller obtained the highest tracking accuracy ($E_{x}$: $5.1930\times 10^{-7}$, $E_{y}$: $2.4647\times 10^{-7}$, and $E_{z}$: $7.9830\times 10^{-7}$) among four control methods, as shown in Figure 3, Figure 4 and Figure 5 and Figure 8, and Table 4. Overall, all three controllers, including SMC, TSMC, and FTSMC, have proven their effectiveness in trajectory tracking when they could provide relatively high tracking accuracy with the respective accuracy of each method, as follows: SMC ($E_{x}$: $1.8896\times 10^{-4}$, $E_{y}$: $3.3387\times 10^{-4}$, and $E_{z}$: $3.5264\times 10^{-4}$), TSMC ($E_{x}$: $2.4920\times 10^{-5}$, $E_{y}$: $4.5702\times 10^{-5}$, and $E_{z}$: $5.3520\times 10^{-5}$), and FTSMC ($E_{x}$: $2.3561\times 10^{-5}$, $E_{y}$: $3.9127\times 10^{-5}$, and $E_{z}$: $5.1999\times 10^{-5}$).

**Figure 9:**
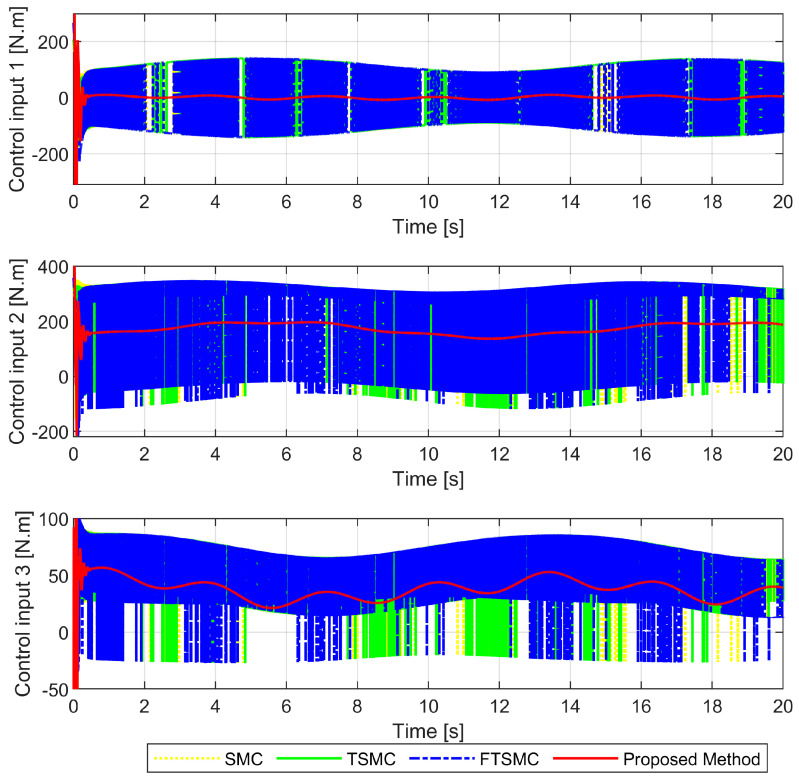
Analysis of chattering behaviors among the four approaches with their respective control signals.

In a chattering comparison, as shown in Figure 9, we recognized that only the proposed controller seems to have provided a continuous control torque without loss of robustness because it applied a reaching control law based on SOSMC, while the three remaining controllers provided high-frequency control torques (which is known as a chattering occurrence in the control signals) because those methods applied a reaching control law based on FOSMC. With high-frequency control torques, those three controllers could guarantee the robustness to cope with the effects of uncertain elements and maintain the tracking accuracy. However, the result of chattering, which appears as dangerous system vibrations, means that moving parts in actuators and mechanical actuators are often subjected to unnecessary wear and tear [20].

## 5. Conclusions

We presented a modified prescribed performance-tracking control system with finite-time convergence stability for URMs. Closed-loop dynamics were improved in several respects, such as estimated convergence speed and a predefined upper and lower limit for maximum overshoot during transient responses. In addition, the maximum allowable size of the control errors at the steady-state could be predefined, and these errors inevitably converge to zero within a finite time, while the proposed controller could provide a smooth control torque without the loss of its robustness. It has been shown that the proposed control system was globally stable and convergent within a finite time. Through the simulation of an industrial robot manipulator, the proposed control algorithm has been proven to be capable of manipulating the convergence speed and the maximum overshoot of the state error variables during the transient response process. In addition, the proposed controller with a combination of the designed nonlinear sliding-mode surface and the STM could attain high tracking accuracy for state error variables in a steady state.

In future work, we intend to make two proposals: (1) a modified performance function which has an explicit pre-specified terminal time, and (2) a full consideration of the influence of the measured data, in particular in terms of the sensor noise and errors associated with it, via an advanced fixed-time prescribed performance controller. 

## Figures and Tables

**Table 1:** 3-DoF manipulator system parameters.

Description	Link 1	Link 2	Link 3
Link Length (m)	$l_{1}=0.25$	$l_{2}=0.7$	$l_{3}=0.6$
Link Weight (kg)	$m_{1}=33.429$	$m_{2}=34.129$	$m_{3}=15.612$
Center of Mass (mm)	$\begin{array}{c} l_{c1x}=0\\ l_{c1y}=0\\ l_{c1z}=-0.7461 \end{array}$	$\begin{array}{c} l_{c2x}=0.3477\\ l_{c2y}=0\\ l_{c2z}=0 \end{array}$	$\begin{array}{c} l_{c3x}=0.3142\\ l_{c3y}=0\\ l_{c3z}=0 \end{array}$
Inertia (kg.m${}^{2}$)	$\begin{array}{c} I_{1xx}=0.7486\\ I_{1yy}=0.5518\\ I_{1zz}=0.5570 \end{array}$	$\begin{array}{c} I_{2xx}=0.3080\\ I_{2yy}=2.4655\\ I_{2zz}=2.3938 \end{array}$	$\begin{array}{c} I_{3xx}=0.0446\\ I_{3yy}=0.7092\\ I_{3zz}=0.7207 \end{array}$

**Table 2:** Assumed uncertain terms in simulation process.

Assumed Uncertainty Type	Functions
Assumed Dynamic Uncertainties	$\Delta M\left(p\right)=0.2M\left(p\right)$
	$\Delta C\left(p,\dot{p}\right)=0.2C\left(p,\dot{p}\right)$
	$\Delta G\left(p\right)=0.2G\left(p\right)$
Assumed Frictions $F_{r}\left(\dot{p}\right)\;\left(N.m\right)$	$F_{r1}\left(\dot{p}\right)=0.1\mathrm{sgn}\left({\dot{p}}_{1}\right)+2{\dot{p}}_{1}$
	$F_{r2}\left(\dot{p}\right)=0.1\mathrm{sgn}\left({\dot{p}}_{2}\right)+2{\dot{p}}_{2}$
	$F_{r3}\left(\dot{p}\right)=0.1\mathrm{sgn}\left({\dot{p}}_{3}\right)+2{\dot{p}}_{3}$
Assumed Disturbances $\tau_{d}\left(t\right)\;\left(N.m\right)$	$\tau_{d1}\left(t\right)=4\sin(t)$
	$\tau_{d2}\left(t\right)=5\sin(t)$
	$\tau_{d3}\left(t\right)=6\sin(t)$

**Table 3:** The proposed controller parameters.

Description	Symbol	Value
PPF in Equation (12)	$\delta_{l},\delta_{u},\mu$, $\psi_{0}$, $\psi_{\infty }$	1,1,10, ${\left[0.34,0.15,0.30\right]}^{T}$, ${\left[0.001,0.001,0.001\right]}^{T}$
Sliding Surface in Equation (22)	$\theta ,h,j,\rho_{1},\rho_{2}$	3,3,2,50,10
Proposed Method in Equation (26)	$\varepsilon ,\alpha_{10},\alpha_{20},\alpha_{30},\alpha_{40}$	3,2,6,10,100

**Table 4:** RMS error with investigation time ranges from 6 s → 20 s via four controllers.

Control System	$E_{x}$	$E_{y}$	$E_{z}$
SMC [2]	$\begin{array}{c} 1.8896\times 10^{-4} \end{array}$	$\begin{array}{c} 3.3387\times 10^{-4} \end{array}$	$\begin{array}{c} 3.5264\times 10^{-4} \end{array}$
TSMC [33]	$\begin{array}{c} 2.4920\times 10^{-5} \end{array}$	$\begin{array}{c} 4.5702\times 10^{-5} \end{array}$	$\begin{array}{c} 5.3520\times 10^{-5} \end{array}$
FTSMC [33]	$\begin{array}{c} 2.3561\times 10^{-5} \end{array}$	$\begin{array}{c} 3.9127\times 10^{-5} \end{array}$	$\begin{array}{c} 5.1999\times 10^{-5} \end{array}$
Proposed Controller	$\begin{array}{c} 5.1930\times 10^{-7} \end{array}$	$\begin{array}{c} 2.4647\times 10^{-7} \end{array}$	$\begin{array}{c} 7.9830\times 10^{-7} \end{array}$

## Data Availability

The data sets generated and/or analyzed during the current study are available from the corresponding author upon reasonable request.

## Abbreviations

The following abbreviations are used in this manuscript:

SM	Sliding Mode
SMC	Sliding-Mode Control
TSM	Terminal Sliding Mode
TSMC	Terminal Sliding-Mode Control
FTSMC	Fast Terminal Sliding-Mode Control
INTSM	Integral Non-Singular Terminal Sliding Mode
FOSMC	First-Order Sliding-Mode Control
SOSM	Second-Order Sliding Mode
SOSMC	Second-Order Sliding-Mode Control
HOSMC	Higher-Order Sliding-Mode Control
FnTC	Finite-Time Control
FxTC	Fixed-Time Control
STM	Super-Twisting Method
PFC	Prescribed Performance Control
PPF	Prescribed Performance Function
DoF	Degrees of Freedom
BsCM	Back-Stepping Control Method
RMS	Root Mean Square
URM	Uncertain Robotic Manipulator
CAD	Computer-Aided Design
